# Parvovirus enteritis and other risk factors associated with persistent gastrointestinal signs in dogs later in life: a retrospective cohort study

**DOI:** 10.1186/s12917-022-03187-7

**Published:** 2022-03-11

**Authors:** Kanae Sato-Takada, Anne M. Flemming, Maarten J. Voordouw, Anthony P. Carr

**Affiliations:** 1 Department of Small Animal Clinical Sciences, Western College of Veterinary Medicine, University of Saskatchewan, Saskatoon, SK Canada; 2 Central Animal Hospital, Kamloops, BC Canada; 3 Department of Veterinary Microbiology, Western College of Veterinary Medicine, University of Saskatchewan, SK Saskatoon, Canada

**Keywords:** Antimicrobials, Canine, Diarrhea, Gastrointestinal system, Immunology, Metoclopramide, Parvovirus, Vomiting

## Abstract

**Background:**

Parvoviral enteritis (PE) is a viral gastrointestinal (GI) infection of dogs. Recovery from PE has been associated with persistent GI signs later in life. The objectives of this study were: (i) To determine whether dogs that have recovered from PE (post-parvo dogs) had an increased risk of persistent GI signs compared to uninfected control dogs. (ii) To investigate the lifestyle and clinicopathologic factors that are associated with persistent GI signs in post-parvo dogs.

**Methods:**

A total of 86 post-parvo dogs and 52 age-matched control dogs were enrolled in this retrospective cohort study. Many years after hospitalization for PE, the owners were interviewed about the health and habits of their dogs using a questionnaire. We used generalized linear mixed effects models to test whether parvovirus enteritis and other risk factors are associated with owner-recognized general health problems in all dogs and with owner-recognized persistent GI signs in post-parvo dogs.

**Results:**

The prevalence of persistent GI signs was significantly higher in post-parvo dogs compared to control dogs (57% vs 25%, *P* < 0.001). Markers of disease severity at the time of hospital admission such as neutropenia, low body temperature (BT), and treatment with an antiemetic medication (metoclopramide) were significant risk factors for persistent GI signs in post-parvo dogs. For example, PE-affected dogs that were hypothermic at hospital admission (BT of 37.2 °C) were 16.6 × more likely to have GI signs later in life compared to hyperthermic dogs (BT of 40.4 °C). The presence of persistent GI signs in post-parvo dogs was a risk factor for health problems in other organ systems.

**Conclusions:**

Parvovirus enteritis is a significant risk factor for persistent GI signs in dogs highlighting the importance of prevention. The risk factors identified in the present study may guide future investigations on the mechanisms that link parvovirus enteritis to chronic health problems in dogs.

**Supplementary Information:**

The online version contains supplementary material available at 10.1186/s12917-022-03187-7.

## Background

Canine parvovirus type 2 (CPV-2) is a non-enveloped, single-stranded DNA virus that is highly contagious among canines. CPV-2 causes parvoviral enteritis (PE), which is characterized by severe gastroenteritis in dogs, usually puppies [1]. The clinical signs of PE include lethargy, vomiting, fever, diarrhea, and neutropenia. These clinical signs are caused by the viral destruction of rapidly dividing cells, including intestinal crypt cells and neutrophils [2]. CPV-2 infects a variety of organs including the small intestine, tonsils, lymph nodes, thymus, spleen, heart, liver, and kidneys [3]. In untreated dogs, PE has a mortality rate of 10–20% [1], but it can be successfully managed by in-hospital or outpatient treatment.

Dogs that recover from parvovirus infection have increased risk of long-term gastrointestinal (GI) signs compared to uninfected control dogs [4], but the factors underlying this increased risk have not been investigated [4]. Microbiome studies on dogs infected with CPV-2 have shown perturbed fecal microbiota compared to uninfected control dogs [5, 6]. These studies have also found that the gut microbiome of dogs infected with CPV-2 has a higher relative abundance of bacteria such as *Campylobacter*, *Bacteroides*, and *Clostridium*, which have been associated with inflammatory bowel disease in dogs [5, 6]. Dogs with PE are frequently treated with antimicrobials to combat secondary bacterial infections, and these treatments will also perturb the gut microbiome [7–11] with unknown consequences for long-term health. Thus, perturbations in the gut microbiome of the dog caused by CPV-2 and/or by antimicrobial treatments during hospitalization might be linked with the development of long-term GI signs.

In humans, there are conflicting results about the long-term consequences of severe diarrhea in early childhood [12–15]. Children infected with non-typhoid *Salmonella* and/or exposed to farms (considered unhygienic environments) were less likely to develop autoimmune diseases (e.g. allergic rhinoconjunctivitis, asthma) later in life [12]. Infants that had diarrhea in their first year of life, mounted a more vigorous immune response to vaccination during adolescence [16]. In contrast, other studies have shown that severe enteritis in infants interferes with their immune systems and may result in food allergies and inflammatory bowel disease [13, 14]. Severe diarrhea in childhood has been associated with physical and cognitive deficits in older children [15]. Bacterial gastroenteritis in adults has been associated with gastrointestinal disorders such as irritable bowel syndrome [17]. Potential mechanisms underlying these long-term consequences of severe diarrhea include changes in GI permeability [18] and perturbations in the gut microbiome [19, 20].

The first aim of our study was to confirm whether dogs that have recovered from PE (post-parvo dogs) are more likely to suffer from long-term GI signs compared to uninfected control dogs. The second aim was to identify the risk factors associated with long-term GI signs in post-parvo dogs. We hypothesized that clinicopathological surrogate markers of disease severity (e.g., degree of neutropenia, treatment with anti-emetics or antibiotics) would be associated with long-term GI signs. Identifying the risk factors that predict whether post-parvo dogs will develop long-term GI signs will improve our treatment of this important disease and enhance our understanding of how acute viral infections during development influence lifetime health.

## Results

### Comparison of explanatory variables between control dogs versus post-parvo dogs

This study contains a total of 138 dogs of which 52 control dogs and 44 post-parvo dogs were obtained in 2011 and an additional 42 post-parvo dogs were obtained in 2019. Hence there are a total of 52 control dogs and 86 post-parvo dogs. The percentage of female dogs was similar between the control group (48.1%; 25/52) and the post-parvo group (47.6%; 41/86). The percentage of purebred dogs was similar between the control group (50.0%; 26/52) and the post-parvo group (47.6%; 41/86).

The mean age at admission was similar between the 2011 control dogs (mean = 27.0 weeks; range = 9.6 – 124.9 weeks), the 2011 post-parvo dogs (mean = 27.3 weeks; range = 6.0 – 106.0 weeks), and the 2019 post-parvo dogs (mean = 33.9 weeks; range = 6.0 – 192.0 weeks). The mean time to follow up was similar between the 2011 control dogs (mean = 7.66 years; range 3.09 – 12.42 years) and the 2011 post-parvo dogs (mean = 7.11 years; range 2.96 – 12.40 years), but it was much shorter for the 2019 post-parvo dogs (mean = 3.34 years; range 0.48 – 8.63 years). The mean age at follow up was similar between the 2011 control dogs (mean = 8.18 years; range 3.57 – 13.59 years) and the 2011 post-parvo dogs (mean = 7.63 years; range 3.42 – 12.61 years), but it was much younger for the 2019 post-parvo dogs (mean = 4.00 years; range 0.75 – 9.09 years). Due to the differences in the mean time to follow up and the mean age at follow between the 2011 and the 2019 samples, it was important to include these two explanatory variables in all statistical analyses.

### Comparison of the prevalence of persistent GI signs between control dogs versus post-parvo dogs

The prevalence of persistent GI signs at follow up in post-parvo dogs (57.0% = 49/86) was significantly higher (2.3x) compared to the control dogs (25.0% = 13/52; χ^2^ = 12.13, df = 1, *P* < 0.001). However, this simple comparison does not consider the many other factors that can influence the probability of persistent GI signs at follow up (see below). We point out that our questionnaire did not ask the owners to evaluate the persistence of GI signs. Instead, we use the term ‘persistent GI signs at follow up’ to indicate the fact that the owners recognized GI signs in their post-parvo dogs many years after these dogs were hospitalized for PE. For consistency, we also use the term ‘persistent GI signs at follow up’ for the control dogs that exhibited GI signs (as recognized by the owner at follow up) many years after they were admitted to the hospital for reasons other than PE.

### Explanatory variables and response variables

The 26 explanatory variables and the 7 response variables used in the statistical analyses are shown in Table 1. The explanatory variables are either factors (i.e., categorical variables with levels) or covariates (i.e., measured on an integer scale or a continuous scale). The response variables are all binomial in nature and refer to whether the dog owners recognized clinical signs in the 6 different organ systems of their dogs (ears, gastrointestinal system, orthopedic system, respiratory system, skin, and urinary system). For simplicity, we refer to the owner-recognized clinical signs in the 6 different organ systems of the dogs as ‘signs’. These 33 variables were obtained from the hospital records when the dogs were hospitalized (both post-parvo and control dogs), from the complete blood count (CBC) panel (only post-parvo dogs during hospitalization for PE), and from the questionnaire (both post-parvo and control dogs). Definitions of the variables derived from the questionnaire are given in Sect. 2 of the supplementary material.

**Table 1:** List of explanatory variables and response variables that were used in the statistical analyses. The explanation of the columns is as follows. Type 1 refers to whether the variable is an explanatory variable or a response variable. There were 26 explanatory variables and 7 response variables. Type 2 refers to whether the explanatory variable is a factor (i.e., consists of levels or categories) or a covariate (is measured on an integer scale or a continuous scale). Type 2 also indicates that the 7 response variables are binomial in nature. Levels or units refers to the levels (or categories) of the factors or the units of measurement of the covariates. Origin refers to whether the variable was obtained from the hospital records, the complete blood count (CBC) panel, or the questionnaire. Definitions of the variables derived from the questionnaire are given in Sect. 2 of the supplementary material. The response variable ‘Clinical signs in 5 systems’ (variable 33 in Table 1) sums the signs over 5 organ systems (i.e., it does not include clinical signs in the GI system) and therefore ranges from 0 to 5

Rank	Variable	Type 1	Type 2	Levels or units	Origin
1	Parvovirus infection history	Explanatory	Factor	Control, Post-parvo	Hospital
2	Organ system	Explanatory	Factor	Ear, GI, Ortho, Resp, Skin, Urinary	Questionnaire
3	Sex	Explanatory	Factor	Female, Male	Hospital
4	Purebred	Explanatory	Factor	Mixed, Purebred	Hospital
5	Lifestyle	Explanatory	Factor	Indoors, In & out, Outdoors	Questionnaire
6	Vaccination	Explanatory	Factor	No, Yes	Questionnaire
7	Deworming	Explanatory	Factor	No, Yes	Questionnaire
8	Medical history	Explanatory	Factor	No, Yes	Questionnaire
9	Age of dog at admission	Explanatory	Covariate	Days	Hospital
10	Time of follow up	Explanatory	Covariate	Days	Questionnaire
11	Weight of dog at admission	Explanatory	Covariate	Kilograms	Hospital
12	Metoclopramide	Explanatory	Factor	No, Yes	Hospital
13	Number of antiemetics	Explanatory	Covariate	0—4	Hospital
14	Number of prescribed antacids	Explanatory	Covariate	0—3	Hospital
15	Number of prescribed antimicrobials	Explanatory	Covariate	0—10	Hospital
16	Duration of hospitalization	Explanatory	Covariate	Hours	Hospital
17	Body temperature of dog at admission	Explanatory	Covariate	degrees Celsius	Hospital
18	Total white blood cell count	Explanatory	Covariate	10^9 cells per litre	CBC panel
19	Segmented neutrophil count	Explanatory	Covariate	10^9 cells per litre	CBC panel
20	Banded neutrophil count	Explanatory	Covariate	10^9 cells per litre	CBC panel
21	Lymphocyte count	Explanatory	Covariate	10^9 cells per litre	CBC panel
22	Eosinophil count	Explanatory	Covariate	10^9 cells per litre	CBC panel
23	Basophil count	Explanatory	Covariate	10^9 cells per litre	CBC panel
24	Monocyte count	Explanatory	Covariate	10^9 cells per litre	CBC panel
25	Hematocrit	Explanatory	Covariate	Proportion of RBCs in blood	CBC panel
26	Toxic change	Explanatory	Covariate	0—4	CBC panel
27	Clinical signs in ears	Response	Binomial	No, Yes	Questionnaire
28	Clinical signs in GI system	Response	Binomial	No, Yes	Questionnaire
29	Clinical signs in orthopedic system	Response	Binomial	No, Yes	Questionnaire
30	Clinical signs in respiratory system	Response	Binomial	No, Yes	Questionnaire
31	Clinical signs in skin	Response	Binomial	No, Yes	Questionnaire
32	Clinical signs in urinary system	Response	Binomial	No, Yes	Questionnaire
33	Clinical signs in 5 systems	Response	Binomial	0—5	Questionnaire

### Analysis of factors that influence general organ signs in control dogs and post-parvo dogs

**Fig. 1:**
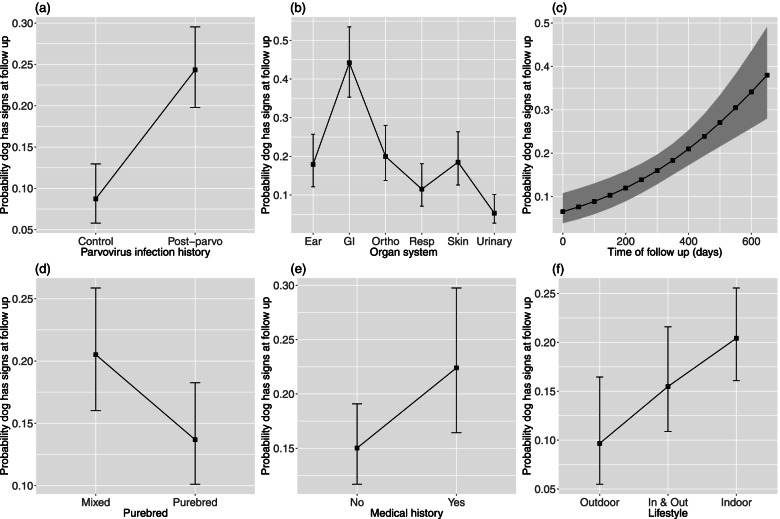
Risk factors for persistent health problems for control and post-parvo dogs. Effects of 6 explanatory variables on the probability that the dogs have signs in the 6 organ systems at the time of follow up. The 6 explanatory variables are as follows: (**a**) parvovirus infection history (Control, Post-parvo), (**b**) organ system (Ear, GI, Orthopedic, Respiratory, Skin, Urinary), (**c**) time of follow up with owners (days), (**d**) purebred (Mixed, Purebred), (**e**) medical history (No, Yes), and (**f**) lifestyle (Outdoor, Indoor and Outdoor, Indoor). The sample size included 52 control dogs and 86 post-parvo dogs (total of 138 dogs). The y-axis shows the probability that the dogs will develop signs in the 6 organ systems at the time of follow up. Shown are the estimated marginal means (EMMs) and their 95% confidence intervals (CIs). To facilitate interpretation, the continuous variables are shown on the x-axis in their original units rather than in units of standard deviation

We used a generalized linear mixed effects model (GLMM) with binomial errors to analyze whether an individual dog experienced signs in the 6 organ systems at follow up (0 = no signs, 1 = signs; response variables 27 – 32 in Table 1). For this analysis, there were 52 control dogs and 86 post-parvo dogs (total of 138 dogs). The complete statistical analysis is provided in Sect. 4 of the supplementary material. For GLMMs with binomial errors, the parameter estimates are measured on the logit scale (see Table S2 in the supplementary material). To determine the effect of each explanatory variable, we calculated the estimated marginal means (EMMs; measured on the logit scale) and then converted these values to the original probability scale (ranging from 0.00 to 1.00). Thus, Fig. 1 and Table 2 show the effects of the explanatory variables of interest on the probability that the control dogs and post-parvo dogs experienced signs at follow up in the 6 organ systems.

**Table 2:** Estimated marginal means (EMMs) of the probability that the 6 organ systems in the dogs have owner-reported signs at follow up. The sample size includes 52 control dogs and 86 post-parvo dogs. Explanatory variables consist of factors with levels or covariates that are measured on a continuous scale that has units. For example, the factor ‘Parvovirus infection’ has two levels ‘Control’ and ‘Post-parvo’; the covariate ‘Time of Follow up’ is measured in units of days. For each row the estimated marginal mean (EMM), standard error (SE), and the lower limit (LL) and upper limit (UL) of the 95% confidence interval are shown

Variable	Levels	Units	EMM	SE	95% LL	95% UL
Parvovirus Infection	Control	NA	8.7	22.6	5.8	13.0
Parvovirus Infection	Post-parvo	NA	24.3	13.5	19.8	29.5
Organ System	Ear	NA	17.9	23.4	12.1	25.7
Organ System	GI	NA	44.2	19.0	35.3	53.5
Organ System	Orthopedic	NA	20.0	22.7	13.8	28.0
Organ System	Respiratory	NA	11.5	27.0	7.1	18.1
Organ System	Skin	NA	18.5	23.2	12.6	26.4
Organ System	Urinary	NA	5.3	35.6	2.7	10.1
Time of Follow up	0	Days	6.5	28.1	3.9	10.8
Time of Follow up	100	Days	8.9	22.0	5.9	13.0
Time of Follow up	200	Days	12.0	16.7	8.9	15.9
Time of Follow up	300	Days	16.0	12.9	12.9	19.7
Time of Follow up	400	Days	21.0	12.4	17.2	25.3
Time of Follow up	500	Days	27.1	15.3	21.5	33.4
Time of Follow up	600	Days	34.1	20.3	25.8	43.5
Purebred	Mixed	NA	20.5	15.4	16.0	25.9
Purebred	Purebred	NA	13.7	17.5	10.1	18.3
Medical History	No	NA	15.0	14.7	11.7	19.1
Medical History	Yes	NA	22.4	19.6	16.4	29.8
Lifestyle	Outdoor	NA	9.7	31.1	5.5	16.5
Lifestyle	In & Out	NA	15.5	20.8	10.9	21.6
Lifestyle	Indoor	NA	20.4	14.9	16.1	25.6

After model simplification, 8 of the 11 explanatory variables remained in the model, of which 6 were significant (Fig. 1; Sect. 4 of the supplementary material). The estimated marginal mean (EMM) probability of signs in the 6 organ systems at follow up was 2.8 × higher in the post-parvo dogs (EMM = 24.3%) compared to the control dogs (EMM = 8.7%; Fig. 1a; Table 2; *P* < 0.001). The EMM probability of signs in the ears, the GI system, the orthopedic system, the respiratory system, the skin, and the urinary system were 17.9%, 44.2%, 20.0%, 11.5%, 18.5%, and 5.3%, respectively (Fig. 1b; Table 2). Using the ears as a reference group, the probability of signs at follow up was significantly higher in the GI tract (*P* < 0.001) and significantly lower in the urinary system (*P* = 0.001). The EMM probability of signs in the 6 organ systems at follow up was positively associated with the time of follow up (Fig. 1c; *P* < 0.001). For example, the EMM probability of signs in the 6 organ systems for a follow up time of 600 days (34.1%) was 3.8 × higher compared to a follow up time of 100 days (8.9%; Table 2). The EMM probability of signs in the 6 organ systems at follow up was 1.5 × higher in the mixed breed dogs (EMM = 20.5%) compared to the purebred dogs (EMM = 13.7%; Fig. 1d; Table 2; *P* = 0.023). The EMM probability of signs in the 6 organ systems at follow up was 1.5 × higher in dogs with a medical history (EMM = 22.4%) compared to dogs with no medical history (EMM = 15.0%; Fig. 1e; Table 2; *P* = 0.035). The EMM probability of signs in the 6 organ systems at follow up was 2.1 × higher in indoor dogs (EMM = 20.4%) compared to outdoor dogs (EMM = 9.7%; Fig. 1f; Table 2; *P* = 0.009).

### Comparison of explanatory variables between post-parvo dogs without GI signs versus the post-parvo dogs with GI signs

The post-parvo dogs that were included in the analysis (*n* = 60) received the following treatments during their hospitalization: 23.3% (14/60) were given metoclopramide, 35.0% (21/60) were given antiemetics (mean = 0.50; range = 0 – 3 antiemetics per dog), 25.0% (15/60) were given antacids (mean = 0.27; range = 0 – 2 antacids per dog), and 98.3% (59/60) were given antimicrobials (mean = 1.53; range = 0 – 4 antimicrobials per dog), with ampicillin being the most common type of antimicrobial.

### Analysis of the factors that influence persistent GI signs at follow up in post-parvo dogs

**Fig. 2:**
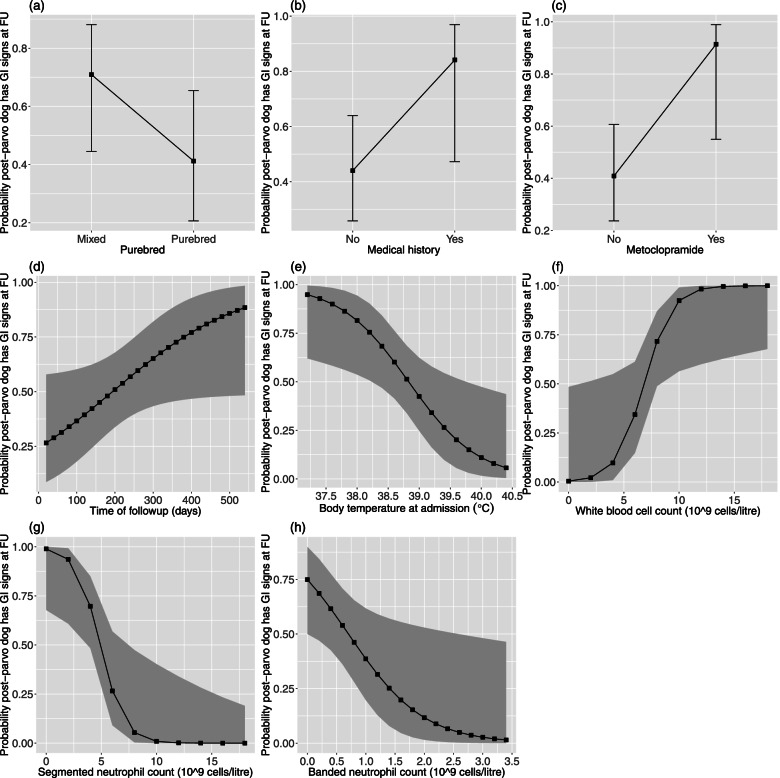
Risk factors for persistent GI signs in post-parvo dogs. Effects of 8 explanatory variables on the probability that the post-parvo dogs had persistent GI symptoms at follow up (FU). The 8 explanatory variables are as follows: (**a**) purebred (Mixed, Purebred), (**b**) medical history (No, Yes), (**c**) metoclopramide treatment (No, Yes), (**d**) time of follow up with owners (days), (**e**) body temperature at admission (°C), (**f**) white blood cell count (10^9 cells per litre of blood), (**g**) segmented neutrophil count (10^9 cells per litre of blood), and (**h**) banded neutrophil count (10^9 cells per litre of blood). The sample size included 31 and 29 post-parvo dogs with and without persistent GI signs, respectively (total of 60 post-parvo dogs). The y-axis shows the probability that the post-parvo dogs will develop persistent GI signs at follow up (FU). Shown are the EMMs and their 95% CIs . To facilitate interpretation, the continuous variables are shown on the x-axis in their original units rather than in units of standard deviation

We used a generalized linear model (GLM) with binomial errors to investigate the variables associated with persistent GI signs in the post-parvo dogs (response variable 28 in Table 1). For this analysis, there were 31 and 29 post-parvo dogs with and without persistent GI signs, respectively (total of 60 post-parvo dogs). The complete statistical analysis is provided in Sect. 6 of the supplementary material. For GLMs with binomial errors, the parameter estimates are measured on the logit scale (see Table S8 in the supplementary material). To determine the effect of each explanatory variable, we calculated the EMMs (measured on the logit scale) and then converted these values to the original probability scale (ranging from 0.00 to 1.00). Thus, Fig. 2 and Table 3 show the effects of the explanatory variables of interest on the probability that the post-parvo dogs experienced persistent GI signs at follow up.

**Table 3:** Estimated marginal means (EMMs) of the probability that the gastrointestinal system in the post-parvo dogs have owner-reported signs at follow up. The sample size includes 60 post-parvo dogs. Explanatory variables consist of factors with levels or covariates that are measured on a continuous scale that has units. For example, the factor ‘Purebred’ has two levels ‘Mixed’ and ‘Purebred’; the covariate ‘Body Temperature’ is measured in units of °C. For each row the estimated marginal mean (EMM), standard error (SE), and the lower limit (LL) and upper limit (UL) of the 95% confidence interval are shown

Variable	Levels	Units	EMM	SE	95% LL	95% UL
Purebred	Mixed	NA	70.9	56.7	44.5	88.1
Purebred	Purebred	NA	41.2	50.6	20.6	65.4
Medical History	No	NA	44.0	41.6	25.8	64.0
Medical History	Yes	NA	84.1	90.6	47.3	96.9
Metoclopramide	No	NA	40.9	40.9	23.7	60.7
Metoclopramide	Yes	NA	91.4	110.4	55.0	98.9
Time of Follow up	80	Days	33.9	53.5	15.3	59.4
Time of Follow up	160	Days	45.1	39.1	27.6	63.9
Time of Follow up	240	Days	56.8	37.3	38.7	73.2
Time of Follow up	320	Days	67.7	49.4	44.4	84.7
Time of Follow up	400	Days	77.0	68.4	46.8	92.8
Time of Follow up	480	Days	84.3	90.0	47.9	96.9
Body temperature	37.2	°C	94.9	123.8	62.0	99.5
Body temperature	38	°C	81.5	68.2	53.7	94.4
Body temperature	38.8	°C	51.3	36.9	33.9	68.5
Body temperature	39.6	°C	20.2	73.9	5.6	51.8
Body temperature	40.4	°C	5.7	130.2	0.5	43.6
White blood cell	0	10^9 cells/litre	0.5	271.1	0.0	48.5
White blood cell	4	10^9 cells/litre	9.8	123.6	1.0	54.9
White blood cell	8	10^9 cells/litre	71.7	49.7	48.9	87.0
White blood cell	12	10^9 cells/litre	98.3	187.4	60.0	100.0
White blood cell	16	10^9 cells/litre	99.9	336.4	65.4	100.0
Segmented neutrophil	0	10^9 cells/litre	98.9	193.3	67.8	100.0
Segmented neutrophil	4	10^9 cells/litre	69.7	45.9	48.4	85.0
Segmented neutrophil	8	10^9 cells/litre	5.4	140.8	0.4	47.4
Segmented neutrophil	12	10^9 cells/litre	0.1	300.8	0.0	33.9
Segmented neutrophil	16	10^9 cells/litre	0.0	462.8	0.0	23.3
Banded neutrophil	0	10^9 cells/litre	74.9	55.8	50.0	89.9
Banded neutrophil	0.8	10^9 cells/litre	46.2	40.4	28.0	65.5
Banded neutrophil	1.6	10^9 cells/litre	19.8	82.6	4.7	55.5
Banded neutrophil	2.4	10^9 cells/litre	6.6	136.7	0.5	50.9
Banded neutrophil	3.2	10^9 cells/litre	2.0	193.0	0.0	47.2

After model simplification, 8 of the 24 explanatory variables remained in the model, of which 5 were significant (Fig. 2; Sect. 6 of the supplementary material). The EMM probability of persistent GI signs at follow up was 2.2 × higher in dogs treated with metoclopramide during hospitalization (EMM = 91.4%) compared to dogs not treated with metoclopramide during hospitalization (EMM = 40.9%; Fig. 2c; Table 3; *P* = 0.027). The probability of persistent GI signs at follow up was negatively associated with the body temperature of the dog at hospital admission (Fig. 2e; *P* = 0.019). For example, the probability of persistent GI signs at follow up was 16.6 × higher in post-parvo dogs with an admission body temperature of 37.2 °C (EMM = 94.9%) compared to post-parvo dogs with an admission body temperature of 40.4 °C (EMM = 5.7%; Table 3). Thus, post-parvo dogs with higher body temperatures (fever) at admission were less likely to have persistent GI signs at follow up. Total white blood cell count was positively associated with persistent GI signs at follow up (Fig. 2f; *P* = 0.037), whereas counts of segmented neutrophils (Fig. 2g; *P* = 0.023) and counts of banded neutrophils (Fig. 2h; *P* = 0.031) were negatively associated with persistent GI signs at follow up. Thus, dogs with more severe neutropenia (i.e., low counts of neutrophils) at admission were more likely to have persistent GI signs at follow up.

The three explanatory variables that were not statistically significant in the above analysis (*n* = 60) are mentioned here because they were significant in another analysis with a larger sample size (*n* = 79), but that did not include the CBC variables (see Sect. 5 of the supplementary material). The probability of persistent GI signs was positively associated with the time of follow up (Fig. 2d; *P* = 0.054). For example, the EMM probability of persistent GI signs for a follow up time of 480 days (84.3%) was 2.5 × higher compared to a follow up time of 80 days (33.9%; Table 3). The EMM probability of persistent GI signs at follow up was 1.7 × higher in mixed breed dogs (EMM = 70.9%) compared to purebred dogs (EMM = 41.2%; Fig. 2a; Table 3; *P* = 0.108). The EMM probability of persistent GI signs at follow up was 1.9 × higher in dogs with a medical history (EMM = 84.1%) compared to dogs with no medical history (EMM = 44.0%; Fig. 2b; Table 3; *P* = 0.064).

### Comparison of general signs between post-parvo dogs with or without persistent GI signs

**Fig. 3:**
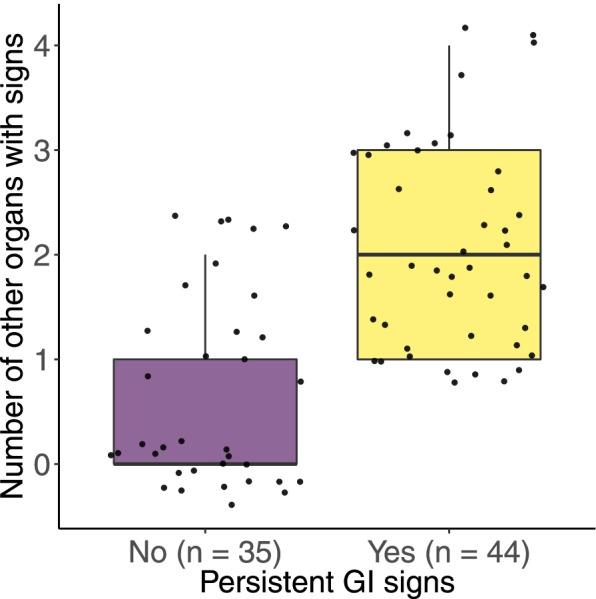
Post-parvo dogs with persistent GI signs have other health problems. Post-parvo dogs with persistent GI symptoms (*n* = 44) have more symptoms in the other 5 organ systems compared to post-parvo dogs with no persistent GI symptoms (*n* = 35). The 5 organ systems include ear, orthopedic, respiratory, skin, and urinary system. The y-axis shows the number of organ systems with signs at the time of follow up. The boxplots show the median (black line), 25th and 75th percentiles (edges of the box), and minimum and maximum values (whiskers). The individual data points (black dots) were jiggered for each dog to visualize their distribution

For the sample of 79 post-parvo dogs, we compared whether post-parvo dogs with persistent GI signs were more likely to have signs in the other 5 organ systems (*n* = 44 dogs with 220 organ systems) compared to post-parvo dogs without persistent GI signs (*n* = 35 dogs with 175 organ systems). The prevalence of signs in the other 5 organ systems (response variable 33 in Table 1) was 1.6 × higher in post-parvo dogs with persistent GI signs (21.4% = 47/220) compared to post-parvo dogs without persistent GI signs (Fig. 3; 13.1% = 23/175). A GLMM with binomial errors that analyzed the prevalence of signs in the other 5 organs as a function of 17 explanatory variables confirmed that this difference between dogs with persistent GI signs versus dogs without persistent GI signs was significant (see Sect. 7 of the supplementary material).

## Discussion

### Parvovirus enteritis is a risk factor for persistent GI signs in dogs

Our study confirmed that dogs that recover from PE are more likely to suffer from persistent GI signs at follow up compared to control dogs. The prevalence of persistent GI signs at follow up in the post-parvo dogs was 57%, which was 2.3 times higher compared to the age-matched control dogs (25%; Fig. 1). Our results are consistent with two other studies that found an association between PE and the presence of persistent GI signs in post-parvo dogs [4]. Our study found a higher prevalence of persistent GI signs (57%) in post-parvo dogs compared to a recent study by another research group (42%) [4]. Explanations for this discrepancy include differences in the questionnaire (e.g., number and types of questions), differences in how the responses of the owners were converted to whether the dog has persistent GI signs or not, and differences in the time of follow up. Both studies show that a high percentage of dogs have persistent GI signs after recovery from parvovirus infection, and it is therefore important to identify the underlying risk factors for these chronic GI health problems.

### Risk factors that influence organ signs in control and post-parvo dogs

There were numerous risk factors that influenced the probability of whether the control dogs and post-parvo dogs had signs at the time of follow up in the 6 organ systems surveyed by the questionnaire. The probability of signs in the 6 organ systems was significantly associated with the time of follow up (Fig. 1). The time of follow up ranged from 0.48 to 12.42 years, where the maximum represents a significant fraction of the lifespan of the average dog. This result was expected because a longer time to follow up means that the dog is older at the time of the questionnaire and has therefore had more time to develop the diseases and signs associated with old age [21–23]. Our study shows the importance of controlling for the time of follow up by including it as a covariate in the statistical analysis. The shorter time of follow up for the post-parvo dogs compared to the control dogs (i.e., due to the inclusion of the 2019 sample, which contained only post-parvo dogs and which had a shorter time of follow up than the 2011 sample), suggests that our study underestimates the effect of PE on future health problems (i.e., because the post-parvo dogs were younger and therefore expected to be healthier at the time of follow up compared to the control dogs).

At follow up we found that owners were significantly more likely to report signs for the GI tract (44.2%) and significantly less likely to report signs for the urinary system (5.3%) compared to the reference organ system (ears; 17.9%; Fig. 1). Parvovirus enteritis is foremost a disease of the GI tract and so we expect GI signs to be more common at follow up compared to signs in other organ systems. Another explanation is that our questionnaire asked more questions about the GI tract (6 questions) compared to the other 5 organ systems (2 – 3 questions per organ system). All else being equal, the probability of detecting at least one sign for a given organ system increases with the number and types of questions targeting that organ system. Thus, it is not surprising that our questionnaire found a higher prevalence of signs for the GI tract compared to the other 5 organ systems. Differences in the number and types of questions (essentially a measure of sampling effort) may also explain why the prevalence of signs was lower in the urinary system compared to the other five organ systems.

Dogs with a medical history (i.e., treatment with other medications at any point in time during the period of follow up) were significantly more likely (1.5x) to have signs in the 6 organ systems at follow up compared to dogs with no medical history (Fig. 1). This result is expected because dogs with health problems are more likely to be treated with medication, and their owners are more likely to report these dogs as having signs on the questionnaire. Due to the variety of medications prescribed for different health problems, we did not investigate whether any medication was associated with signs in the 6 organ systems of the dogs. In summary, dogs that are treated with medication during the time of follow up are more likely to be reported by their owners as having signs at the time of follow up.

Our study found that indoor dogs were significantly more likely (2.1x) to have signs in the 6 organ systems at follow up compared to outdoor dogs (Fig. 1). One explanation is a human monitoring effect; owners may have more opportunities to monitor the health of indoor dogs compared to outdoor dogs. An interesting alternative explanation is the hygiene hypothesis, which was developed to explain the proliferation of autoimmune diseases (allergies, asthma, etc.) in human populations of the developed world. Numerous studies have found that children that spend more time in the outdoors and/or in unhygienic environments are less likely to develop allergies and autoimmune diseases [12, 16]. Our observation that an indoor lifestyle is a risk factor for persistent health problems in dogs (as recognized by the dog owners) suggests that the hygiene hypothesis may also be true for canids.

Mixed breed dogs were significantly more likely (1.5x) to have signs in the 6 organ systems at follow up compared to purebred dogs (Fig. 1). This effect was unexpected because purebred dogs, which are more inbred, tend to have more health problems than mixed breed dogs, which are outbred [24, 25]. One explanation is differences in exposure to CPV; mixed breed dogs are more likely to come from shelters where the risk of exposure to CPV is much higher compared to purebred dogs, which come from carefully controlled breeding kennels [26]. Vaccination coverage against CPV is lower in shelters compared to breeding kennels, which influences the level of maternally derived antibody (MDA) titres in pups [26]. MDA titres are important for protecting pups against CPV; pups with intermediate and low versus absent CPV-specific MDA titres developed mild versus severe disease, respectively [27]. Thus, purebred mothers are more likely to be vaccinated and the MDA titres in purebred pups are more likely to protect them from severe disease compared to mixed breed mothers and their pups. Finally, owners of expensive purebred dogs might be more pro-active at bringing their PE-affected pets to the hospital compared to the owners of mixed breed dogs. Earlier hospitalization and intervention would prevent PE from becoming too severe and would reduce the probability of PE-associated chronic health problems in purebred dogs compared to mixed breed dogs. In summary, mixed breed dogs are expected to have higher exposure to CPV, lower levels of protective antibodies to prevent severe disease, and may experience delayed or lower quality treatment compared to purebred dogs. More severe disease in mixed breed dogs would result in more PE-associated health problems later in life compared to purebred dogs.

### Risk factors that influence persistent GI signs in post-parvo dogs

Our study suggest that dogs that are more severely ill with PE are at greater risk of developing persistent GI signs later in life. For the post-parvo dogs, there was a significant negative association between the body temperature at hospital admission and persistent GI signs at the time of follow up (Fig. 2). The probability of post-parvo dogs having persistent GI signs at the time of follow up was 16.6 × times higher for hypothermic dogs (body temperature of 37.2 °C) compared to hyperthermic dogs (body temperature of 40.4 °C). Previous studies on PE in dogs have suggested that hypothermia at the time of hospital admission indicates severe metabolic disease or shock [2, 28, 29]. Hypothermia in PE-affected dogs at the time of hospital admission is a marker of disease severity, which in turn appears to be an important risk factor for developing persistent GI signs later in life.

Our study also found that neutropenia during hospital admission was significantly associated with a higher probability of persistent GI signs at follow up (Fig. 2). Neutropenia (low neutrophil count in blood) is a known clinical sign of PE and is consistent with the pathology of PE. CPV-2 targets the rapidly dividing precursor cells in the bone marrow that produce neutrophils, which results in neutropenia [2, 30]. Our observation that neutropenia is a risk factor for persistent GI signs, suggest that low neutrophil counts are a marker of severe PE. In contrast, total WBC counts during hospitalization for PE were positively associated with persistent GI signs at follow up. Besides neutrophils, the total WBC count includes leucocytes such as lymphocytes, eosinophils, basophils, and monocytes. The reason for the positive association between WBC counts and the probability of persistent GI signs at follow up is uncertain as it cannot be attributed to a consistent increase in another leukocyte type given the lack of significance found when the individual leukocyte values were analyzed.

In the analysis of the probability of signs in the 6 organ systems at follow up for the control dogs and the post-parvo dogs, the explanatory variables of purebred status, medical history, and the time of follow up were all significant (Fig. 1). The same result was found in an analysis of the probability of persistent GI signs at follow up for a larger sample of post-parvo dogs (*n* = 79), which did not include the explanatory variables from the CBC panel (Sect. 5 of the supplementary material). However, when the explanatory variables from the CBC panel were included, the sample size decreased (*n* = 60), and these 3 explanatory variables were no longer statistically significant (Fig. 2). The explanations as to why purebred status, medical history, and time of follow up are risk factors for persistent GI signs in post-parvo dogs are probably the same explanations as to why these three explanatory variables are risk factors for signs in the 6 organ systems in all dogs (control and post-parvo).

### Metoclopramide treatment is associated with persistent GI signs in post-parvo dogs

For the post-parvo dogs, the use of metoclopramide during hospitalization for PE was the only treatment associated with an increased risk of persistent GI signs (Fig. 2). The reasons for this association are speculative. Although metoclopramide is an antiemetic, it is not considered a very effective treatment at our institution and is therefore rarely used for this purpose. We believe that metoclopramide was used at our institution whenever ileus was considered a major component of vomiting. Unfortunately, it is not possible to retrospectively determine the reasons why clinicians used metoclopramide. Ileus severe enough to require intervention may also be a marker of disease severity, though this too is speculative. In critically ill children, secondary ileus can lead to bacterial overgrowth [31], and in canine PE patients, gut bacterial translocation is linked with septic complications [2].

Our study found no evidence that the use of multiple antiemetics was a risk factor for persistent GI signs. We expected that dogs with severe vomiting during their hospitalization for PE would be given more antiemetics and therefore that the use of multiple antiemetics would be a marker for disease severity. This type of bias is common in retrospective studies where sicker patients get more treatments than less sick patients, which results in the treatments being associated with poorer health outcomes. For example, in a retrospective study on parvovirus treatment, dogs given antiemetics had longer hospitalization times than dogs that were not given antiemetics [32]. The reason why our study did not find the expected positive association between the number of antiemetics prescribed and the probability of persistent GI signs at follow up is unclear. A well-defined prospective study would be required to determine whether vomiting and ileus are markers for the severity of PE.

### Use of multiple antimicrobials are not a risk factor for persistent GI signs in post-parvo dogs

The use of multiple antimicrobials during hospital admission was not associated with persistent GI signs in post-parvo dogs at the time of follow up. This result was surprising because in our institution, multiple antimicrobials are routinely used in dogs that are assessed as more severely affected, whereas a single antimicrobial such as ampicillin is generally used in those dogs that are less ill. Studies on human patients have shown that the alteration of the gut microbiota depends on the type of antimicrobial used [20]. For this reason, we performed a more detailed statistical analysis on the three antimicrobials (ampicillin, amikacin, and gentamycin) that were most frequently used to treat PE-affected dogs in our study (supplementary material, Sect. 5). This analysis found that these three antimicrobials had no significant effects on the probability of persistent GI signs in post-parvo dogs at the time of follow up. We expected that the use of antimicrobials could permanently alter the intestinal microbiota of dogs with lasting consequences for their health, as such effects have been shown in mice and humans [19, 33]. However, this expectation is contradicted by studies that have shown that the gut microbiota of dogs returns to its original state in 2 to 4 weeks after the cessation of antimicrobial treatment [8–10]. Other studies have shown that the gut microbiome in dogs with PE is substantially different compared to healthy, uninfected dogs [5, 6]. However, it remains to be determined whether these PE-induced changes in the gut microbiome persist over the lifetime of the dog and whether they are responsible for the persistent GI signs in post-parvo dogs at the time of follow up observed in the present study.

### Persistent GI signs is a risk factor for other health problems in post-parvo dogs

Post-parvo dogs with persistent GI signs were 1.6 times more likely to have signs in the other 5 organ systems (ear, orthopedic, respiratory, skin, and urinary) that were assessed with the questionnaire. Previous studies on humans have found that severe diarrhea experienced in childhood is a risk factor for health problems in other organ systems during adulthood [13–15, 17]. Our study suggests that PE during puppyhood is a risk factor for a variety of health problems for post-parvo dogs later in life.

### Limitations of the study

The present retrospective study has several limitations. One limitation is that treatment protocols were at the discretion of the attending clinician (i.e., they were not standardized) and therefore we do not know the decisions underlying the various treatment regimens. Standardized management plans for dogs with PE do not exist at our institution. Similarly, there were no objective measures by the clinician of the severity of PE in the dogs (e.g., frequency of vomiting, frequency of diarrhea, weight loss, degree of dehydration, presence of ileus). Another limitation is the subjectivity of the owners in assessing the health status of their dogs at the time of follow up. Thus, two dogs with the same clinical signs might be classified differently according to the perceptions of their owners. Conversely, two dogs with different clinical signs might be classified as being the same according to the perceptions of their owner. However, a strong defense of our study is that the hospital measures of disease severity (e.g., body temperature, neutrophil count, and metoclopramide treatment), of which the owners were not aware, were strongly associated with owner assessments of dog health, which occurred an average of 6.2 years after hospitalization for PE. To create such associations through bias, the owners would have to somehow know that their dog had a severe case of PE and then remember to exaggerate the health symptoms of their dog accordingly in a follow up interview many years later. We therefore conclude that the risk factors for persistent GI symptoms in post-parvo dogs identified in this study are biologically real rather than imagined by their owners.

## Conclusion

More than half of the dogs that recovered from PE suffered from persistent GI signs later in life. Some clinical factors in post-parvo dogs such as time to follow up, indoor lifestyle, body temperature at hospital admission, use of metoclopramide, WBC counts, and neutrophil counts were risk factors for persistent GI signs. Persistent GI signs in post-parvo dogs are common, and it is therefore important to investigate the underlying mechanisms. Our study shows the importance of owner education and preventive vaccination against CPV-2 to protect puppies from developing persistent GI problems later in life.

## Methods

### Study aims

(i) To determine whether dogs that have recovered from PE (post-parvo dogs) had an increased risk of persistent GI signs compared to uninfected controls. (ii) To investigate the lifestyle and clinicopathologic factors that are associated with persistent GI signs in post-parvo dogs.

### Study design

This is a retrospective cohort study. Client-owned dogs that had been diagnosed and treated for PE (post-parvo dogs) at the teaching hospital of the Western College of Veterinary Medicine at the University of Saskatchewan from April 1999 to December 2018 were identified using the medical record system. The diagnosis of PE was based on appropriate history and clinical signs, and a positive point-of-care (POC) ELISA kit test for canine parvovirus antigen (SNAP® Canine Parvovirus Antigen Test Kit, IDEXX Laboratories, Inc., Westbrook, Maine, USA). Control dogs were selected using the same medical record system and were matched to post-parvo dogs using two criteria: (i) the control dog was presented for vaccination within 2 weeks of admission of the post-parvo dog and (ii) the control dog was within 6 months of the age of the post-parvo dog. These two criteria prevented us from matching the dog breed between the control dogs and the post-parvo dogs.

Several years after hospital admission, the owners of the dogs (control and post-parvo) were contacted by phone to complete our questionnaire, which included basic questions about the lifestyle and health of the dog. Owners were asked to report only on signs that occurred after the PE hospitalization and during the follow up period. Dogs with completed questionnaires were included in our retrospective study, and the duration between hospital admission and the phone interview was recorded as the time of follow up. We completed questionnaires for 52 control dogs and 44 post-parvo dogs in 2011. To increase the sample size, we completed questionnaires for an additional 42 post-parvo dogs in 2019. Thus, our total sample size is 52 control dogs and 86 post-parvo dogs.

### Questionnaire

The questionnaire is available in Sect. 1 of the supplementary material, and it contained 31 questions regarding the current health status of the dog. The questionnaire addressed the following health conditions: presence of persistent GI signs, vomiting, diarrhea, owners’ perception of “sensitive stomach”, signs consistent with pruritus of skin or ear, ear infection, respiratory signs, orthopedic signs, urinary tract disease signs, weight loss or gain, polyuria-polydipsia, vaccination status, and deworming status. The questionnaire also included information on the diet history, length of the feeding period for the current diet, lifestyle (indoor, outdoor), and medical history other than parvoviral enteritis, which was defined as the dog being treated with medications in the time interval between the end of the original hospital admission (for PE or vaccination) and the questionnaire interviews (i.e., medications related to treatment for PE were not included).

Whenever appropriate, owners were asked to assess the degree of clinical signs. We used the information from the questionnaire to classify dogs (control and post-parvo) as having GI signs (yes versus no) depending on whether the owners recognized the signs of vomiting and/or diarrhea (at least 1 sign versus 0 signs). Similarly, we used the questionnaire to classify the dogs as having signs for the 5 other organ systems: ear, orthopedic, respiratory, skin, and urinary (Sect. 2 of the supplementary material).

### Clinicopathological data

Clinicopathological data were extracted from electronic medical records and included hospitalization data, in-hospital management data, and laboratory data. The hospitalization data included age at admission (weeks), breed, and gender. The in-hospital management data included use and type of antiemetics (e.g., maropitant, ondansetron, metoclopramide, etc.), use and type of antimicrobials (e.g., ampicillin, amikacin, and gentamycin, etc.), and use and type of antacids (e.g., H_2_ blocker, proton-pump inhibitor). The laboratory data included the complete blood count (CBC) panel with blood smear evaluation by clinical pathologists. For dogs where the CBC panel was performed more than once, the replicate panel with the lowest leukocyte count was selected for the statistical analysis.

### Statistical analysis

All the statistical analyses were done using R version 1.3.959. The details of the statistical methods are given in Sect. 3 of the supplementary material. A *P* value of less than 0.05 is considered statistically significant.

### Analysis of factors that influence general organ signs in control dogs and post-parvo dogs

We used a GLMM with binomial errors to analyze whether an individual dog experienced signs in the 6 organ systems at follow up (0 = no signs, 1 = signs; response variables 27 – 32 in Table 1; see Sect. 4 of the supplementary material for details). The identity of the dog was modelled as a random factor to account for non-independence of the 6 organ systems from the same dog. There were 11 explanatory variables (Table 1): (1) parvovirus infection history (control, post-parvo), (2) organ system (ear, GI, orthopedic, respiratory, skin, and urinary system), (3) sex (female, male), (4) purebred (mixed, purebred), (5) lifestyle (indoors only, indoors and outdoors, outdoors only), (6) up-to-date vaccination (no, yes), (7) deworming treatment given (no, yes), (8) medical history (no, yes), (9) age of dog at admission (days), (10) time of follow up (days), and (11) weight of dog at admission (kg). The continuous variables were transformed to z-scores (mean of 0.0, standard deviation of 1.0) to facilitate model convergence and comparison of the effect size between variables measured in different units. We simplified the model by sequentially removing explanatory variables with a *P* value > 0.10. For this analysis, there were 52 control dogs and 86 post-parvo dogs (total of 138 dogs).

### Analysis of the factors that influence persistent GI signs at follow up in post-parvo dogs

We used a GLM with binomial errors to determine the variables associated with persistent GI signs at follow up in the post-parvo dogs (response variable 28 in Table 1; see Sect. 6 of the supplementary material for details). There were 24 explanatory variables: 15 from the questionnaire and in-hospital management and 9 from the CBC panel (Table 1). The 15 explanatory variables from the questionnaire and in-hospital management were as follows: (1) sex (female, male), (2) purebred (mixed, purebred), (3) lifestyle (indoors only, indoors and outdoors, outdoors only), (4) up-to-date vaccination (no, yes), (5) deworming treatment given (no, yes), (6) medical history (no, yes), (7) age of dog at admission (days), (8) time of follow up (days), (9) weight of dog at admission (kg), (10) metoclopramide treatment (no, yes), (11) number of prescribed of antiemetics (0 – 4), (12) number of prescribed antacids (0 – 3), (13) number of prescribed antimicrobials (0 – 10), (14) duration of hospitalization (hours), and (15) dog body temperature at admission (°C). The 9 explanatory variables from the CBC panel were as follows: (1) total white blood cells, (2) segmented neutrophils, (3) banded neutrophils, (4) lymphocytes, (5) eosinophils, (6) basophils, (7) monocytes, (8) hematocrit, and (9) toxic change. As before, the continuous variables were transformed to z-scores, and we simplified the model by sequentially removing explanatory variables with a *P* value > 0.10. For this analysis, there were 31 and 29 post-parvo dogs with and without persistent GI signs, respectively (total of 60 post-parvo dogs).

## Supplementary Information

**Additional file 1.** Supplementary material 

## Data Availability

The datasets generated and/or analysed during the current study are not publicly available due to limitations of ethical approval involving the patient data and anonymity but are available from the corresponding author on reasonable request. Requests for the datasets should be directed to Maarten J. Voordouw (maarten.voordouw@usask.ca).

## Abbreviations

CBC: Complete blood count
CPV-2: Canine parvovirus type-2
GI: Gastrointestinal
PE: Parvoviral enteritis
POC: Point-of-care
GLMM: Generalized linear mixed effects model
GLM: Generalized linear model
