# Supplementary material for: Parvovirus enteritis and other risk factors associated with persistent gastrointestinal signs in dogs later in life: a retrospective cohort study
Source: BMC Vet Res. 2022 Mar 11;18:96. doi: 10.1186/s12917-022-03187-7 (PMC8915519; doi:10.1186/s12917-022-03187-7)
Supplement: Supplementary file 1 — Additional file 1. Supplementary material [file 12917_2022_3187_MOESM1_ESM.docx]

Supplementary Material

Title: Parvovirus enteritis and other risk factors associated with persistent gastrointestinal signs in dogs later in life: A retrospective cohort study

Authors: Kanae Takada, Anne M. Flemming, Maarten J. Voordouw, and Anthony P. Carr

Table of Contents

[Section 1 – Questionnaire given to the clients about their dogs 2](#_Toc91679261)

[Section 2 – Creation of response variables and explanatory variables from the questionnaire 4](#_Toc91679262)

[Section 3 – General statistical methods 5](#_Toc91679263)

[Section 4 – Analysis of general organ signs in control dogs and post-parvo dogs (n = 138 dogs) 6](#_Toc91679264)

[Section 5 – Analysis of persistent GI signs in post-parvo dogs; explanatory variables taken from questionnaire and hospital management (n = 79 post-parvo dogs) 9](#_Toc91679265)

[Section 6 – Analysis of persistent GI signs in post-parvo dogs; explanatory variables taken from questionnaire, hospital management and the CBC panel (n = 60 post-parvo dogs) 12](#_Toc91679266)

[Section 7 – Analysis of general organ signs in post-parvo dogs with or without persistent GI signs (n = 80 post-parvo dogs) 16](#_Toc91679267)

[Section 8 – Dog breeds used in the study 19](#_Toc91679268)

# Section 1 – Questionnaire given to the clients about their dogs

[Agreement to answer the questionnaire]

Do you still own (animal’s name)?

If no 🡪 is the dog deceased?

If the dog has died 🡪 Would you be willing to answer some questions about his/her health?

[Environmental information]

- Is (animal’s name) an inside or an outside dog?
- Do you consider your dog to be healthy?
- What kind of food do you feed your dog?
- How long have you fed this food? If food has been switched, why?
- Do you give your dog;
- Treats or cookies?
- Rawhides?
- Vitamins?
- Any “people food”?

[Weight gain/loss, vaccination/deworming histories, medications]

- Has a veterinarian ever told you that your dog might need to gain weight or lose weight?
- Is your dog currently on any medication? If so, for what condition?
- Has your dog been treated by a veterinarian in the past for any serious condition other than regular check-ups and vaccinations? (for parvo dogs, ask “only after being treated for parvovirus”)
- Has your dog received regular vaccinations? When was the last vaccination?
- Has your dog ever reacted to the vaccinations? If so, what happened?
- Has your dog ever been given dewormer?
- Has your dog ever been on medication for any condition? (only ask this if they are not currently on meds.) If so, do you recall what the medication was and what it was for?

[Skin signs]

- Does your dog ever seem to be especially itchy? (No = 0)

If so, would you say it is:

__every now and then (mild=1)/__every day (moderate=2)/

__multiple times a day or all the time (severe = 3)

- Are there any areas on your dog where there is missing hair/ flaky skin/ redness?
- Has your dog had any problems with its toenails or feet? If so, what kind of problems?

[Ear signs]

- Has your dog ever had any problems with its ears? If so, what kind of problems?
- Does your dog ever chew on its feet? (No = 0)

If so, would you say it is:

__ every now and then (mild = 1)/__ every day (moderate = 2)/

__ multiple times a day / all the time (severe = 3)

- Does your dog shake its head a lot? (No = 0)

If so, would you say it is:

__ every now and then (mild = 1)/__ every day (moderate = 2)/

__ multiple times a day/all the time (severe = 3)

[GI signs]

- Has your dog ever had stomach problems?
- Do you recall any times the dog has had diarrhea or loose stool?

What were the circumstances?

- Do you recall any times the dog has vomited more than once in a day?

How often/how many times?

Did it vomit up digested or undigested food?

- Would you say your dog has a sensitive stomach?
- Does your dog seem to have more gas when it eats a particular food?

If so, what food is it?

- Is there any food that doesn’t seem to agree with him/her?

[Respiratory signs]

- Has your dog ever had a problem with one of the following:
- coughing
- wheezing
- nasal discharge
- weeping eyes

If so, what were the circumstances?

- Has your dog ever been diagnosed with
- a “cold”
- a chest infection
- asthma
- pneumonia
- any other respiratory problem

If so, what were the circumstances?

[Urinary signs]

- Does your dog ever have any “accidents” in the house?

If so, was it urine or feces?

If feces, was it diarrhea? (If diarrhea, count as GI signs)

- Does your dog drink more water than it used to, or want to go outside to urinate more often?

[Orthopedic signs]

- Does your dog ever:
- have difficulty standing up
- seem stiff in its joints
- Has your dog been diagnosed with any problems in its joints, muscles, or ligaments?
- Do you have any other concerns about your dog’s health that we haven’t discussed already?

# Section 2 – Creation of response variables and explanatory variables from the questionnaire

The questionnaire contained 31 questions, of which 18 asked about signs in the 6 organ systems: ear (n = 3 questions), gastrointestinal (n = 6 questions), orthopedic (n = 2 questions), respiratory (n = 2 questions), skin (n = 3 questions), and urinary system (n = 3 questions). The answers from the owners were recorded and converted into the decision of whether each of 6 organ systems tested positive for “signs” at the time of the follow-up phone interview. When specific conditions were recognized in a dog (i.e., pneumonia), the organ signs were considered positive although by the time of phone interview the dog may have recovered.

**Response variables – clinical signs in organ systems of the dogs:** The ear signs were considered positive if the dog had more than mild signs of shaking its head, or scratching its ears, redness of the ear, ear discharge, or diagnosis of ear infection. The ear signs were not considered positive when the owner was not sure whether the frequency or severity of scratching was truly more than expected for a normal dog. The gastrointestinal signs were considered positive if vomiting, diarrhea, or both were noted. The gastrointestinal signs were considered negative if there was only one episode of vomiting or soft stool following dietary indiscretion (i.e., excessive feeding, or fed steak bone), or one episode of vomiting following the rapid consumption of food. The dog was considered to have a “sensitive stomach” if the dog vomited after certain events, such as after eating certain foods or treats, or after stressful events. The orthopedic signs were considered positive if the owner reported previously diagnosed conditions mentioned to the owner during the questionnaire (i.e., difficulty getting up or using stairs, stiffness, etc.). Respiratory signs were considered positive if the owner reported previously diagnosed specific diseases mentioned to the owner during the questionnaire (i.e., coughing, pneumonia, asthma, etc.). Skin signs were considered positive if the owner reported ongoing itchiness, skin redness, chewing at the paw, etc. The urinary signs were considered positive if the owner reported conditions such as polyuria-polydipsia (including one due to diuretic use) and urinary accidents in the house.

**Explanatory variables:** The purebred status of the dog (purebred, mixed) was determined by the owner in response to the question “*Is your dog purebred or not?*”. The lifestyle status of the dog (indoors only, indoors, and outdoors, outdoors only) was determined by the owner in response to the question “*Is (animal’s name) an inside or an outside dog?*”. The up-to-date vaccination status of the dog (no, yes) was determined by the owner in response to the question “*Has your dog received regular vaccinations?*”. The deworming treatment status of the dog (no, yes) was determined by the owner in response to the question “*Has your dog ever been given dewormer?*”. The medical history status of the dog (no, yes) was determined as ‘yes’ if the owner gave a positive response to one of the two following questions “*Is your dog currently on any medication?*” and “*Has your dog ever been on medication for any condition?*”.

# Section 3 – General statistical methods

**GLMs and GLMMs with binomial errors:** The prevalence of signs is a binomial variable; dogs with and without signs were given a 1 or a 0, respectively. We analysed this binomial response variable using generalized linear models (GLMs) with binomial errors or Generalized Linear Mixed effects Models (GLMMs) with binomial errors. GLMs with binomial errors were used if each dog occurred only once in the analysis, which was the case for the analysis of the prevalence of persistent GI signs at follow up. GLMMs with binomial errors were used when signs at follow up were analysed for multiple organ system from the same dog to account for non-independence of repeated measures on the same animal. For all the GLMMs, the dog ID was used as the random effect.

**Model simplification:** Each statistical analysis started with the full model that contained all the explanatory variables of interest. We simplified the models by sequentially removing explanatory variables that were the least statistically significant. After removing an explanatory variable, the model was updated, and the next least significant explanatory variable was selected for removal. Model simplification continued until all explanatory variables had a p-value of < 0.100. These p-values were calculated using type II log-likelihood ratio (LLR) tests. LLR tests compare nested models that differ with respect to the variable of interest. The change in deviance between the two models follows a Chi-square distribution, which is used to calculate the p-values.

**Parameter estimates:** We determined the effect of the explanatory variables by investigating the sign and magnitude of the parameter estimates. In GLMs or GLMMs with binomial errors, the parameter estimates are calculated on the logit scale. To back-calculate these parameter estimates to the original scale of probabilities (or percentages) requires the following transformation: P = exp(Y)/(1 + exp(Y)), where Y is the predicted value (based on the parameter estimates) on the logit scale. R calculates the statistical significance of each parameter estimate by dividing it by its standard errors, which gives a z-statistic that can be used to calculate a p-value. The p-values of the LLR tests (based on Chi-square distribution) and the p-values of the parameter estimates (based on the z-statistic) are generally similar, they are not always identical. The p-values from the LLR tests are generally more sensitive (i.e., more statistically significant) compared to the p-values from the parameter estimates. In the manuscript, we present the more conservative p-values from the parameter estimates. A *P* value < 0.05 is considered statistically significant.

**Effect sizes of explanatory variables:** When a model contains numerous explanatory variables (i.e., a multiple regression model), each parameter estimate is a partial regression coefficient, which indicates the effect of the explanatory variable on the response variable, when all other explanatory variables are held constant. To visualize the effects of the explanatory variables, we used the ggeffects() package in R to calculate the marginal effects at the mean (MEM) or at representative values (MER) from statistical models, i.e. predictions generated by a model when one holds the non-focal variables constant and varies the focal variable(s). The effects of the explanatory variables are shown on the original probability scale.

**R software functions and packages:** All the statistical analyses were done using R version 1.2.5019. The *glm()* function in the base package and the *glmer()* function in the lme4 package were used to create the GLMs and the GLMMs, respectively. The *Anova()* function in the car package was used to generate the p-values from the type II LLR tests; these p-values were used to simplify the model by the sequential removal of non-significant explanatory variables. We used the *ggeffect()* function in the ggeffects package to calculate the marginal effects at the mean and these were plotted using the *ggplot()* function in the ggplot2 package. Finally, we used the *ggarrange()* function in the ggpubr package to create multi-panel plots.

# Section 4 – Analysis of general organ signs in control dogs and post-parvo dogs (n = 138 dogs)

**Statistical Methods:** We used a GLMM with binomial errors to analyze whether an individual dog experiences signs for a given organ system (0 = no signs, 1 = signs). The identity of the dog was modelled as a random factor to account for non-independence of organ signs for the same dog. There were 11 explanatory variables: (1) parvoviral infection history (control, post-parvo), (2) organ system (ear, gastro-intestinal, orthopedic, respiratory, skin, and urinary system), (3) sex (female, male), (4) purebred (no, yes), (5) lifestyle (indoors only, indoors and outdoors, outdoors only), (6) up-to-date vaccination (no, yes), (7) deworming treatment given (no, yes), (8) medical history (no, yes), (9) age of the dog at admission (days), (10) time of follow up (days), and (11) dog weight at admission (kg). The continuous variables were transformed to z-scores (mean of zero, units of standard deviations) to facilitate comparison of the effect size between variables measured in different units. We simplified the model by sequentially removing non-significant explanatory variables. For this analysis, there were 52 control dogs and 86 post-parvo dogs (total of 138 dogs).

**Results:** The residuals of the simplified model met the assumptions of the GLMM, as the ratio of the residual deviance to the residual degrees of freedom was < 1.000 (res dev/res df = 745.7/813 = 0.917). After model simplification, 8 of the 11 explanatory variables remained in the model, of which 6 were significant according to the LLR tests in Table S1: parvoviral infection history (χ^2^ = 23.153, df = 1, p < 0.001), organ system (χ^2^ = 59.588, df = 5, p < 0.001), time of follow up (χ^2^ = 23.204, df = 1, p < 0.001), purebred (χ^2^ = 5.168, df = 1, p = 0.023), medical history (χ^2^ = 4.439, df = 1, p = 0.035), lifestyle (χ^2^ = 7.340, df = 2, p = 0.025), sex (χ^2^ = 3.312, df = 1, p = 0.069), and deworming treatment (χ^2^ = 2.842, df = 1, p = 0.092).

We investigated the parameter estimates in Table S2 to determine the direction and magnitude of the effect of each explanatory variable. The effects of the explanatory variables are shown in Figure S1, which is identical to Figure 1 in the main manuscript. Post-parvo dogs were significantly more likely to have signs than control dogs (Figure S1; Post-parvo - Control contrast = 1.211, SE = 0.252, z = 4.812, p < 0.001). Using the ear as the reference organ, dogs (both control and post-parvo) had significantly more signs in the GI tract (Figure S1; GI - Ear contrast = 1.278, SE = 0.293, z = 4.357, p < 0.001) and significantly fewer signs in the urinary system (Figure S1; Urinary - Ear contrast = -1.364, SE = 0.406, z = -3.356, p = 0.001). The probability of signs was positively associated with the time of follow up (Figure S1; slope = 0.548, SE = 0.114, z = 4.817, p < 0.001). Purebred dogs had significantly fewer signs than mixed breed dogs (Figure S1; Purebred - Mixed contrast = -0.486, SE = 0.214, z = -2.273, p = 0.023). Dogs with a previous medical history had significantly more signs than dogs with no previous medical history (Figure S1; Yes - No contrast = 0.485, SE = 0.230, z = 2.107, p = 0.035). Indoor dogs had significantly more signs than outdoor dogs (Figure S1; Indoor - Outdoor contrast = 0.866, SE = 0.331, z = 2.621, p = 0.009).

Table S1. Analysis of the prevalence of general organ signs using a GLMM with binomial errors. The simplified model contained 8 explanatory variables of which 6 were statistically significant. Shown are the results from the type II log-likelihood test: the change in deviance ( Δ Dev), the change in degrees of freedom ( Δ Df), and the p-value. The sample size included 52 control dogs and 86 post-parvo dogs (total of 138 dogs).

| Variable | Δ Dev | Δ Df | P |
| --- | --- | --- | --- |
| Parvoviral infection history | 23.153 | 1 | <0.001 |
| Organ system | 59.588 | 5 | <0.001 |
| Time of follow up | 23.204 | 1 | <0.001 |
| Purebred | 5.168 | 1 | 0.023 |
| Medical history | 4.439 | 1 | 0.035 |
| Lifestyle | 7.340 | 2 | 0.025 |
| Sex | 3.312 | 1 | 0.069 |
| Deworm treatment | 2.842 | 1 | 0.092 |

Table S2. Parameter estimates of the model shown in Table S1. This model analyzed the prevalence of general organ signs using a GLMM with binomial errors. For each explanatory variable, the type of parameter is defined (e.g., intercept, contrast, slope), and the parameter estimate, standard error (SE), z-value, and p-value are shown. The sample size included 52 control dogs and 86 post-parvo dogs (total of 138 dogs).

| Variable | Parameter definition | Estimate | SE | Z | P |
| --- | --- | --- | --- | --- | --- |
| *** | Intercept | -3.274 | 0.516 | -6.349 | <0.001 |
| Parvoviral infection history | Post-parvo - Control | 1.211 | 0.252 | 4.812 | <0.001 |
| Organ system | GI - Ear | 1.278 | 0.293 | 4.357 | <0.001 |
| Organ system | Orthopedic - Ear | 0.125 | 0.309 | 0.406 | 0.685 |
| Organ system | Respiratory - Ear | -0.526 | 0.338 | -1.556 | 0.120 |
| Organ system | Skin - Ear | 0.028 | 0.312 | 0.090 | 0.928 |
| Organ system | Urinary - Ear | -1.364 | 0.406 | -3.356 | 0.001 |
| Time of follow up | Slope | 0.548 | 0.114 | 4.817 | <0.001 |
| Purebred | Purebred - Mixed | -0.486 | 0.214 | -2.273 | 0.023 |
| Medical history | Yes - No | 0.485 | 0.230 | 2.107 | 0.035 |
| Lifestyle | In and out - Outdoor | 0.532 | 0.349 | 1.522 | 0.128 |
| Lifestyle | Indoor - Outdoor | 0.866 | 0.331 | 2.621 | 0.009 |
| Sex | Male - Female | 0.382 | 0.210 | 1.820 | 0.069 |
| Deworm treatment | Yes - No | 0.388 | 0.230 | 1.686 | 0.092 |

Figure S1. Effects of six explanatory variables on the probability that the dogs have signs at the time of follow up. The six explanatory variables are as follows: (a) parvovirus infection status (Control, Post-parvo), (b) organ system (Ear, GI, Orthopedic, Respiratory, Skin, Urinary), (c) time of follow up with owners (days), (d) purebred (Mixed, Purebred), (e) medical history (No, Yes), and (f) lifestyle (Outdoor, Indoor and Outdoor, Indoor). The sample size included 52 control dogs and 86 post-parvo dogs (total of 138 dogs). The y-axis shows the probability that the dogs will develop signs in the 6 organ systems at the time of follow up. Shown are the estimated marginal means (EMMs) and their 95% confidence intervals. To facilitate interpretation, the continuous variables are shown on the x-axis in their original units rather than in units of standard deviation.

# Section 5 – Analysis of persistent GI signs in post-parvo dogs; explanatory variables taken from questionnaire and hospital management (n = 79 post-parvo dogs)

**Statistical Methods:** We used a GLM with binomial errors to investigate the variables associated with persistent GI signs in the post-parvo dogs. There were 15 explanatory variables: (1) sex (male, female), (2) purebred (no, yes), (3) lifestyle (indoors only, indoors and outdoors, outdoors only), (4) up-to-date vaccination (no, yes), (5) deworming treatment given (no, yes), (6) medical history (no, yes), (7) metoclopramide treatment (no, yes), (8) number of prescribed of anti-vomiting agents (0 – 4), (9) number of prescribed antacids (0 – 3), (10) number of prescribed antibiotics (0 – 10), (11) age of the dog at admission (days), (12) time of follow up (days), (13) duration of hospitalization (hours), (14) dog weight at admission (kg), and (15) dog body temperature at admission (°C). The continuous variables (variables 11, 12, 13, 14, and 15) were transformed to z-scores. We simplified the model by sequentially removing explanatory variables with a p-value > 0.10. For this analysis, there were 44 and 35 post-parvo dogs with and without persistent GI signs, respectively (total of 79 post-parvo dogs).

**Results:** The PE-affected dogs that were included in the analysis (n = 79) received the following treatments during their hospitalization: 25.3% (20/79) were given metoclopramide, 38.0% (30/79) were given antiemetics (mean = 0.53; range = 0 – 3 antiemetics per dog), 24.0% (19/79) were given antacids (mean = 0.25; range = 0 – 2 antacids per dog), 96.2% (76/79) were given antimicrobials (mean = 1.51; range = 0 – 4 antimicrobials per dog), with ampicillin being the most common type.

The residuals of the simplified model met the assumptions of the GLM, as the ratio of the residual deviance to the residual degrees of freedom was close to 1.000 (res dev/res df = 79.663/73 = 1.091). After model simplification, 5 of the 15 explanatory variables remained in the model, of which 5 were significant according to the LLR tests in Table S3: purebred (χ^2^ = 12.462, df = 1, p < 0.001), medical history (χ^2^ = 6.420, df = 1, p = 0.011), metoclopramide treatment during hospitalization (χ^2^ = 5.570, df = 1, p = 0.018), time of follow up (χ^2^ = 6.305, df = 1, p = 0.012), and body temperature of the dog at admission (χ^2^ = 9.056, df = 1, p = 0.003).

We investigated the parameter estimates in Table S4 to determine the direction and magnitude of the effect of each explanatory variable. Purebred dogs were significantly less likely to have persistent GI signs than mixed breed dogs (Figure S2; contrast = -1.965, SE = 0.602, z = -3.266, p = 0.001). Dogs with a previous medical history were significantly more likely to have persistent GI signs than dogs with no previous medical history (Figure S2; contrast = 1.817, SE = 0.778, z = 2.335, p = 0.020). Dogs treated with metoclopramide during hospitalization were significantly more likely to have persistent GI signs than dogs not treated with metoclopramide (Figure S2; contrast = 1.668, SE = 0.763, z = 2.187, p = 0.029). The probability of persistent GI signs was positively associated with the time of follow up (Figure S2; slope = 0.741, SE = 0.318, z = 2.332, p = 0.020). The probability of persistent GI signs was negatively associated with the body temperature of the dog at admission (Figure S2; slope = -0.949, SE = 0.349, z = -2.714, p = 0.007). Thus, dogs with high fever at admission were less likely to have persistent GI signs.

**Effects of specific antibiotics:** One criticism of our approach of investigating the effect of antibiotics during hospitalization for PE on persistent GI signs is that we used the number of prescribed antibiotics. It is possible that the type of antibiotic used during hospitalization for PE has a more important effect on persistent GI signs compared the number of prescribed antibiotics. A total of 10 different antibiotics were prescribed for post-parvo dogs during their hospitalization for parvovirus enteritis. Of the 10 antibiotics, only 3 were prescribed sufficiently often that a statistical analysis was possible. The other 7 antibiotic were only prescribed for 1 dog, so there is not sufficient power to determine their effects. The 3 antibiotics of interest were ampicillin, amikacin, and gentamycin that were prescribed to 93.7% (74/79), 30.4% (24/79), and 17.7% (14/79) of the post-parvo dogs, respectively. For the GLM with binomial errors of GI signs in the 79 post-parvo dogs, these 3 antibiotics had no effect on the probability of persistent GI signs (χ^2^ = 0.443, df = 3, p = 0.931). We sequentially removed gentamycin (p = 0.933), ampicillin (p = 0.933), and amikacin (p = 0.933) from the GLM, but none of these antibiotics had a significant effect on the probability of persistent GI signs. In summary, including the identity of these 3 antibiotics had no effect on the GLM of persistent GI signs in the post-parvo dogs.

Table S3. Analysis of the persistent GI signs using a GLM with binomial errors. The simplified model contained 5 explanatory variables of which 5 were statistically significant. Shown are the results from the type II log-likelihood test: the change in deviance ( Δ Dev), the change in degrees of freedom ( Δ Df), and the p-value. The sample size included 44 and 35 post-parvo dogs with and without persistent GI signs, respectively (total of 79 post-parvo dogs).

| Variable | Δ Dev | Δ Df | P |
| --- | --- | --- | --- |
| Purebred | 12.462 | 1 | <0.001 |
| Medical history | 6.420 | 1 | 0.011 |
| Metoclopramide | 5.570 | 1 | 0.018 |
| Time of follow up | 6.305 | 1 | 0.012 |
| Temperature at admission | 9.056 | 1 | 0.003 |

Table S4. Parameter estimates of the model shown in Table S3. This model analyzed the prevalence of persistent GI signs using a GLM with binomial errors. For each explanatory variable, the type of parameter is defined (e.g., intercept, contrast, slope), and the parameter estimate, standard error (SE), z-value, and p-value are shown. The sample size included 44 and 35 post-parvo dogs with and without persistent GI signs, respectively (total of 79 post-parvo dogs).

| Variable | Parameter definition | Estimate | SE | Z | P |
| --- | --- | --- | --- | --- | --- |
| *** | Intercept | 0.465 | 0.440 | 1.057 | 0.290 |
| Purebred | Purebred - Mixed | -1.965 | 0.602 | -3.266 | 0.001 |
| Medical history | Yes - No | 1.817 | 0.778 | 2.335 | 0.020 |
| Metoclopramide | Yes - No | 1.668 | 0.763 | 2.187 | 0.029 |
| Time of follow up | Slope | 0.741 | 0.318 | 2.332 | 0.020 |
| Temperature at admission | Slope | -0.949 | 0.349 | -2.714 | 0.007 |

Figure S2. Effects of five explanatory variables on the probability that the post-parvo dogs has persistent GI signs at follow up (FU). The five explanatory variables are as follows: (a) purebred (Mixed, Purebred), (b) medical history (No, Yes), (c) metoclopramide treatment (No, Yes), (d) time of follow up with owners (days), and (e) body temperature at admission ( °C). The sample size included 44 and 35 post-parvo dogs with and without persistent GI signs, respectively (total of 79 post-parvo dogs). The y-axis shows the probability that the dogs will develop signs in the 6 organ systems at the time of follow up. Shown are the estimated marginal means (EMMs) and their 95% confidence intervals. To facilitate interpretation, the continuous variables are shown on the x-axis in their original units rather than in units of standard deviation.

# Section 6 – Analysis of persistent GI signs in post-parvo dogs; explanatory variables taken from questionnaire, hospital management and the CBC panel (n = 60 post-parvo dogs)

**Statistical Methods:** We used a GLM with binomial errors to investigate the variables associated with persistent GI signs in the post-parvo dogs. There were 15 explanatory variables from the questionnaire and hospital management: (1) sex (male, female), (2) purebred (no, yes), (3) lifestyle (indoors only, indoors and outdoors, outdoors only), (4) up-to-date vaccination (no, yes), (5) deworming treatment given (no, yes), (6) medical history (no, yes), (7) metoclopramide treatment (no, yes), (8) number of prescribed of anti-vomiting agents (0 – 4), (9) number of prescribed antacids (0 – 3), (10) number of prescribed antibiotics (0 – 10), (11) age of the dog at admission (days), (12) time of follow up (days), (13) duration of hospitalization (hours), (14) dog weight at admission (kg), and (15) dog body temperature at admission (°C). There were another 9 explanatory variables from the CBC panel: (1) total WBC, (2) segmented neutrophils, (3) banded neutrophils, (4) lymphocytes, (5) eosinophils, (6) basophils, (7) monocytes, (8) hematocrit, and (9) toxic change. As before, the continuous variables were transformed to z-scores and we simplified the model by sequentially removing explanatory variables with a p-value > 0.10. For this analysis, there were 31 and 29 post-parvo dogs with and without persistent GI signs, respectively (total of 60 post-parvo dogs).

**Results:** The PE-affected dogs that were included in the analysis (n = 60) received the following treatments during their hospitalization: 23.3% (14/60) were given metoclopramide, 35.0% (21/60) were given antiemetics (mean = 0.50; range = 0 – 3 antiemetics per dog), 25.0% (15/60) were given antacids (mean = 0.27; range = 0 – 2 antacids per dog), 98.3% (59/60) were given antimicrobials (mean = 1.53; range = 0 – 4 antimicrobials per dog), with ampicillin being the most common type.

The residuals of the simplified model met the assumptions of the GLM, as the ratio of the residual deviance to the residual degrees of freedom was close to 1.000 (res dev/res df = 50.410/51 = 0.988). After model simplification, 8 of the 24 explanatory variables remained in the model, of which 7 were significant according to the LLR tests in Table S5: purebred (χ^2^ = 2.776, df = 1, p = 0.096), medical history (χ^2^ = 4.078, df = 1, p = 0.043), metoclopramide treatment during hospitalization (χ^2^ = 6.525, df = 1, p = 0.011), time of follow up (χ^2^ = 4.208, df = 1, p = 0.040), body temperature of the dog at admission (χ^2^ = 7.822, df = 1, p = 0.005), white blood cell count (χ^2^ = 5.384, df = 1, p = 0.020), segmented neutrophil count (χ^2^ = 6.536, df = 1, p = 0.011), and banded neutrophil count (χ^2^ = 6.088, df = 1, p = 0.014).

We investigated the parameter estimates in Table S6 to determine the direction and magnitude of the effect of each explanatory variable. The effects of the explanatory variables are shown in Figure S3, which is identical to Figure 2 in the main manuscript. Purebred dogs were less likely to have persistent GI signs than mixed breed dogs (Figure S3; contrast = -1.247, SE = 0.776, z = -1.607, p = 0.108). Dogs with a previous medical history were more likely to have persistent GI signs than dogs with no previous medical history (Figure S3; contrast = 1.907, SE = 1.029, z = 1.853, p = 0.064). Dogs treated with metoclopramide during hospitalization were significantly more likely to have persistent GI signs than dogs not treated with metoclopramide (Figure S3; contrast = 2.733, SE = 1.238, z = 2.207, p = 0.027). The probability of persistent GI signs was positively associated with the time of follow up (Figure S3; slope = 0.843, SE = 0.439, z = 1.923, p = 0.054). The probability of persistent GI signs was negatively associated with the body temperature of the dog at admission (Figure S3; slope = -1.286, SE = 0.546, z = -2.355, p = 0.019). Thus, dogs with high fever at admission were less likely to have persistent GI signs. Total white blood cell count was positively associated with persistent GI signs (Figure S3; slope = 3.643, SE = 1.743, z = 2.091, *P* = 0.037), whereas segmented neutrophils (Figure S3; slope = -4.010, SE = 1.764, z = -2.273, *P* = 0.023) and banded neutrophils (Figure S3; slope = -1.171, SE = 0.543, z = -2.158, *P* = 0.031) were negatively associated with persistent GI signs.

Table S5. Analysis of the persistent GI signs using a GLM with binomial errors. The simplified model contained 8 explanatory variables of which 7 were statistically significant. Shown are the results from the type II log-likelihood test: the change in deviance ( Δ Dev), the change in degrees of freedom ( Δ Df), and the p-value. The sample size included 31 and 29 post-parvo dogs with and without persistent GI signs, respectively (total of 60 post-parvo dogs).

| Variable | Δ Dev | Δ Df | P |
| --- | --- | --- | --- |
| Purebred | 2.776 | 1 | 0.096 |
| Medical history | 4.078 | 1 | 0.043 |
| Metoclopramide | 6.525 | 1 | 0.011 |
| Time of follow up | 4.208 | 1 | 0.040 |
| Temperature at admission | 7.822 | 1 | 0.005 |
| White blood cell count | 5.384 | 1 | 0.020 |
| Segmented neutrophil count | 6.536 | 1 | 0.011 |
| Banded neutrophil count | 6.088 | 1 | 0.014 |

Table S6. Parameter estimates of the model shown in Table S5. This model analyzed the prevalence of persistent GI signs using a GLM with binomial errors. For each explanatory variable, the type of parameter is defined (e.g. intercept, contrast, slope), and the parameter estimate, standard error (SE), z-value, and p-value are shown. The sample size included 31 and 29 post-parvo dogs with and without persistent GI signs, respectively (total of 60 post-parvo dogs).

| Variable | Parameter definition | Estimate | SE | Z | P |
| --- | --- | --- | --- | --- | --- |
| *** | Intercept | -0.254 | 0.601 | -0.423 | 0.672 |
| Purebred | Purebred - Mixed | -1.247 | 0.776 | -1.607 | 0.108 |
| Medical history | Yes - No | 1.907 | 1.029 | 1.853 | 0.064 |
| Metoclopramide | Yes - No | 2.733 | 1.238 | 2.207 | 0.027 |
| Time of follow up | Slope | 0.843 | 0.439 | 1.923 | 0.054 |
| Temperature at admission | Slope | -1.286 | 0.546 | -2.355 | 0.019 |
| White blood cell count | Slope | 3.643 | 1.743 | 2.091 | 0.037 |
| Segmented neutrophil count | Slope | -4.010 | 1.764 | -2.273 | 0.023 |
| Banded neutrophil count | Slope | -1.171 | 0.543 | -2.158 | 0.031 |

Figure S3. Effects of eight explanatory variables on the probability that the post-parvo dogs had persistent GI signs at follow up (FU). The eight explanatory variables are as follows: (a) purebred (Mixed, Purebred), (b) medical history (No, yes), (c) metoclopramide treatment (No, yes), (d) time of follow up with owners (days), (e) body temperature at admission (°C), (f) white blood cell count (10^9 cells per litre of blood), (g) segmented neutrophil count (10^9 cells per litre of blood), and (h) banded neutrophil count (10^9 cells per litre of blood). The sample size included 31 and 29 post-parvo dogs with and without persistent GI signs, respectively (total of 60 post-parvo dogs). The y-axis shows the probability that the post-parvo dogs will develop persistent GI signs. Shown are the estimated marginal means (EMMs) and their 95% confidence intervals. To facilitate interpretation, the continuous variables are shown on the x-axis in their original units rather than in units of standard deviation.

# Section 7 – Analysis of general organ signs in post-parvo dogs with or without persistent GI signs (n = 80 post-parvo dogs)

**Statistical Methods:** We used a GLMM with binomial errors to compare the prevalence of signs in five other organ systems between dogs with or without persistent GI signs. The identity of the dog was modelled as a random factor to account for non-independence of organ signs for the same dog. We included 17 explanatory variables: organ (ear, orthopedic, respiratory, skin, and urinary system), presence of persistent GI signs (no, yes), and the same 15 explanatory variables as above. The sample size was the same as above (total of 80 post-parvo dogs).

**Results:** The residuals of the simplified model met the assumptions of the GLMM, as the ratio of the residual deviance to the residual degrees of freedom was < 1.000 (res dev/res df = 352.1/390 = 0.903). After model simplification, 4 of the 17 explanatory variables remained in the model, of which 4 were significant according to the LLR tests in Table S7: persistent GI signs (χ^2^ = 5.759, df = 1, p = 0.016), organ system (χ^2^ = 8.374, df = 4, p = 0.079), lifestyle (χ^2^ = 5.378, df = 2, p = 0.068), and body temperature of the dog at admission (χ^2^ = 6.842, df = 1, p = 0.009).

We investigated the parameter estimates in Table S8 to determine the direction and magnitude of the effect of each explanatory variable. Post-parvo dogs with persistent GI signs were significantly more likely to have signs in the other 5 organs compared to post-parvo dogs without persistent GI signs (Figure S4; contrast = 0.741, SE = 0.309, z = 2.400, p = 0.016). The prevalence of signs in post-parvo dogs with GI signs (22.2% = 50/225) was 1.7 times higher than that in post-parvo dogs without GI signs (Figure 3; 13.1% = 23/175). Compared to other organs, post-parvo dogs had significantly fewer signs in the urinary system (Figure S4; contrast = -1.167, SE = 0.491, z = -2.375, p = 0.018). Indoor post-parvo dogs had significantly more signs than outdoor post-parvo dogs (Figure S4; contrast = 0.884, SE = 0.384, z = 2.300, p = 0.021). The probability of signs in the 5 other organs was positively associated with the body temperature of the dog at admission (Figure S4; slope = 0.375, SE = 0.143, z = 2.616, p = 0.009). Thus, dogs with high fever at admission were more likely to have signs in other organ systems.

Table S7. Analysis of the prevalence of general organ signs using a GLMM with binomial errors. The simplified model contained four significant explanatory variables. Shown are the results from the type II log-likelihood test: the change in deviance ( Δ Dev), the change in degrees of freedom ( Δ Df), and the p-value. The sample size included 45 and 35 post-parvo dogs with and without persistent GI signs, respectively (total of 80 post-parvo dogs).

| Variable | Δ Dev | Δ Df | P |
| --- | --- | --- | --- |
| GI signs | 5.759 | 1 | 0.016 |
| Organ system | 8.374 | 4 | 0.079 |
| Lifestyle | 5.378 | 2 | 0.068 |
| Temperature at admission | 6.842 | 1 | 0.009 |

Table S8. Parameter estimates of the model shown in Table S7. This model analyzed the prevalence of general organ signs using a GLMM with binomial errors. For each explanatory variable, the type of parameter is defined (e.g., intercept, contrast, slope), and the parameter estimate, standard error (SE), z-value, and p-value are shown. The sample size included 45 and 35 post-parvo dogs with and without persistent GI signs, respectively (total of 80 post-parvo dogs).

| Variable | Parameter definition | Estimate | SE | Z | P |
| --- | --- | --- | --- | --- | --- |
| *** | Intercept | -2.346 | 0.472 | -4.973 | 0.000 |
| GI signs | Yes - No | 0.741 | 0.309 | 2.400 | 0.016 |
| Organ system | Orthopedic - Ear | -0.078 | 0.396 | -0.198 | 0.843 |
| Organ system | Respiratory - Ear | -0.529 | 0.425 | -1.244 | 0.213 |
| Organ system | Skin - Ear | 0.076 | 0.389 | 0.195 | 0.845 |
| Organ system | Urinary - Ear | -1.167 | 0.491 | -2.375 | 0.018 |
| Lifestyle | In and out - Outdoor | 0.571 | 0.432 | 1.319 | 0.187 |
| Lifestyle | Indoor - Outdoor | 0.884 | 0.384 | 2.300 | 0.021 |
| Temperature at admission | Slope | 0.375 | 0.143 | 2.616 | 0.009 |

Figure S4. Effects of four explanatory variables on the probability that the dogs have general organ signs at the time of follow up. The four explanatory variables are as follows: (A) persistent GI signs at follow up, (B) organ system (ear, GI, orthopedic, respiratory, skin, urinary), (C) lifestyle (outdoor, indoor and outdoor, indoor), and (D) body temperature at admission. The sample size included 45 and 35 post-parvo dogs with and without persistent GI signs, respectively (total of 80 post-parvo dogs). The y-axis shows the probability that the post-parvo dogs will develop persistent GI signs.

# Section 8 – Dog breeds used in the study

Table S9. Dog breeds included in the control group and the post-parvo group. The dog breeds are ranked from most abundant to least abundant.

| Dog breed | Control  n (%) | Post-parvo  n (%) |
| --- | --- | --- |
| Mix | 26 (50%) | 44 (52%) |
| Rottweiler | 0 | 5 (5.8%) |
| Boston Terrier | 0 | 3 (3.5%) |
| Border Collie | 0 | 2 (2.4%) |
| Chesapeake Bay Retriever | 0 | 3 (3.5%) |
| Labrador Retriever | 1 (1.9%) | 3 (3.5%) |
| American Pitbull | 1 (1.9%) | 2 (2.4%) |
| German Shepherd Dog | 4 (7.7%) | 2 (2.4%) |
| Golden Retriever | 2 (3.8%) | 2 (2.4%) |
| Shih Tzu | 3 (5.8%) | 0 |
| Spitz | 2 (3.8%) | 1 (1.2%) |
| Chihuahua | 2 (3.8%) | 1 (1.2%) |
| Others | 11 (21.2%) | 17 (19.8%) |
| Total | 52 (100.0%) | 86 (100.0%) |

The control group contained a single individual from the following 11 breeds: Beagle, Bloodhound, Brittany Spaniel, English Springer Spaniel, Jack Russell Terrier, Lhasa Apso, Papillon, Pug, Shetland Sheepdog, Toy poodle, West Highland White Terrier. The post-parvo group contained a single individual from the following 17 breeds: Akita, American Cocker Spaniel, Australian Shepherd, Bulldog, Cane-Corso, English Springer Spaniel, Great Pyrenes, Irish Setter, Miniature Poodle, Norwegian Elkhound, Pomeranian, Pug, Saint Bernard, Siberian Huskey, Shiba Inu, Toy poodle, Weimaraner
